# Nonlinear inference capacity of fiber-optical extreme learning machines

**DOI:** 10.1515/nanoph-2025-0045

**Published:** 2025-06-23

**Authors:** Sobhi Saeed, Mehmet Müftüoğlu, Glitta R. Cheeran, Thomas Bocklitz, Bennet Fischer, Mario Chemnitz

**Affiliations:** Leibniz-Institute of Photonic Technology, Albert-Einstein-Str. 9, 07745 Jena, Germany; Institute of Physical Chemistry, Friedrich Schiller University Jena, Helmholtzweg 4, 07743 Jena, Germany; Institute of Applied Optics and Biophysics, Friedrich Schiller University Jena, Albert-Einstein-Str. 15, 07745 Jena, Germany

**Keywords:** optical neural networks, extreme learning machine, supercontinuum generation, nonlinear fiber optics, optical soliton, machine learning

## Abstract

The intrinsic complexity of nonlinear optical phenomena offers a fundamentally new resource to analog brain-inspired computing, with the potential to address the pressing energy requirements of artificial intelligence. We introduce and investigate the concept of nonlinear inference capacity in optical neuromorphic computing in highly nonlinear fiber-based optical Extreme Learning Machines. We demonstrate that this capacity scales with nonlinearity to the point where it surpasses the performance of a deep neural network model with five hidden layers on a scalable nonlinear classification benchmark. By comparing normal and anomalous dispersion fibers under various operating conditions and against digital classifiers, we observe a direct correlation between the system’s nonlinear dynamics and its classification performance. Our findings suggest that image recognition tasks, such as MNIST, are incomplete in showcasing deep computing capabilities in analog hardware. Our approach provides a framework for evaluating and comparing computational capabilities, particularly their ability to emulate deep networks, across different physical and digital platforms, paving the way for a more generalized set of benchmarks for unconventional, physics-inspired computing architectures.

## 1 Introduction

The rapid advancement of artificial intelligence has sparked renewed interest in brain-inspired hardware, particularly optical implementations that promise energy-efficient solutions for AI acceleration and intelligent edge sensing. Traditional computing architectures face significant challenges when executing analog neural networks, resulting in substantial energy, water, and computational time requirements for operating large networks on conventional digital processors (i.e., CPUs, GPUs, TPUs). Optical approaches have garnered particular attention due to their intrinsic parallelism and scalability across multiple optical degrees of freedom, offering reduced energy consumption [1].

Unlike electronic solutions, a primary challenge in realizing competitive neuromorphic optical hardware lies in the all-optical implementation of nonlinearity to circumvent the electro-optical bottleneck toward deep all-optical architectures [2], [3].

Nonlinearity is crucial for emulating synaptic switching behavior in information networks and enabling advanced learning capabilities, including improved accuracy and generalization. In deep networks, this issue is often addressed through multiple layers, which offer higher nonlinear mapping capabilities of the model. The concept of “nonlinear mapping capability” refers to a system’s ability to transform input data into higher-dimensional spaces where previously inseparable patterns become linearly separable. Moving to hardware, physical substrates can inherently provide complex nonlinear responses through their natural dynamics without the need for additional layers or computational resources.

In optics, taking advantage of the nonlinearity offered by light–matter interactions in the media, while actively studied [4], is widely assumed to be complicated and inefficient due to its high-power demands [5].

Several recent studies suggest alternatives to realize nontrivial nonlinearity in optical systems. These include electronic feedback loops to electro-optic modulators [6], [7], saturable absorption [8] complex active gain dynamics in mode-competitive cavities [9], and, most recently, repeated linear encoding through multiple scattering of input data [10], [11].

Neuromorphic computing with wave dynamics offers a promising approach to the elegant utilization of natural nonlinear dynamics in physical substrates. In optics, the concept aligns with Extreme Learning Machines (ELM) – a form of reservoir computing [12], [13] without internal recurrence. Unlike other optical implementations, the learning machine comprises multiple virtual nodes that are coupled by the intrinsic feed-forward propagation dynamics of a single optical component, such as a fiber. This concept, recently proposed by Marcucci et al. [14], utilizes process-intrinsic, nonlinear mode interactions from natural waveguide dynamics as a computing resource. Experimental demonstrations include nonlinearly coupling spatial modes in multimode fibers [15] and spectral frequency generation in single-mode fibers [16], [17], [18].

However, analog wave computers have a common difficulty: the systems are generally complex and cannot be easily mapped to practical computing models, a critical challenge lies in the diversity of existing approaches and the general inability to measure a system’s nonlinear mapping capabilities independent of specific tasks and to correlate them with learning abilities. This transformation is fundamental to solving complex tasks. Current approaches in the optical community empirically test new nonlinear systems on this capability through task-specific image recognition benchmarks. Here, it is common practice to compare, e.g., accuracies across system configurations to demonstrate improvements and to relate those improvements to the scaling in the learning behavior of deeper, hence more nonlinear neural networks computer models.

In particular, the MNIST dataset is frequently used as a benchmark. Yet, it requires only low nonlinearity to separate the ten classes, as a logistic regression can achieve approximately 92 % accuracy [19]. Similarly, linear Support Vector Machines (SVMs), which leverage kernel tricks to project input data into higher-dimensional spaces for linear separation, demonstrate comparable performance. Furthermore, performance improvements, even if using big networks, cannot be clearly attributed to higher nonlinearity given by the number of hidden layers (i.e., deepness), but might also result from increased connectivity, as we will discuss further down in this paper. The question of nonlinearity’s relevance in neural networks also remains largely unexplored in computer science, with only few investigations into nonbinary nonlinear activation functions, such as a multistep perceptron [20] or trainable splines [21].

This work attempts to better understand and quantify the nonlinear inference capacity using a scalable, task-independent dataset and validate it using two different optical fiber processors. This investigation is particularly relevant for comparing computational capabilities across different physical substrates. We begin with illustrating the operation principle of a frequency-based ELM in a single optical fiber, as an arbitrary kernel machine; we refer to it from this chapter onward as fiber-optical ELM. We then assess the inference capabilities of two different nonlinear systems distinguished by their dispersion properties and corresponding nonlinear dynamics (i.e., self-phase modulation and soliton fission).

We assess the nonlinear inference capacity of the two different fiber-optical machines using a scalable spiral dataset, where increasing the angular span progressively challenges separability. These comparisons were conducted under different system configurations, including variations in fiber types, different numbers of system read-outs, and different power levels. Observations on datasets are compared to digital classifier models, like neural networks and support vector machines, providing a strong basis for drawing parallels between the depth of digital networks and the nonlinear inference capacity of our analog computing systems.

## 2 Materials and methods

### 2.1 Experimental setup

**Figure 1:**
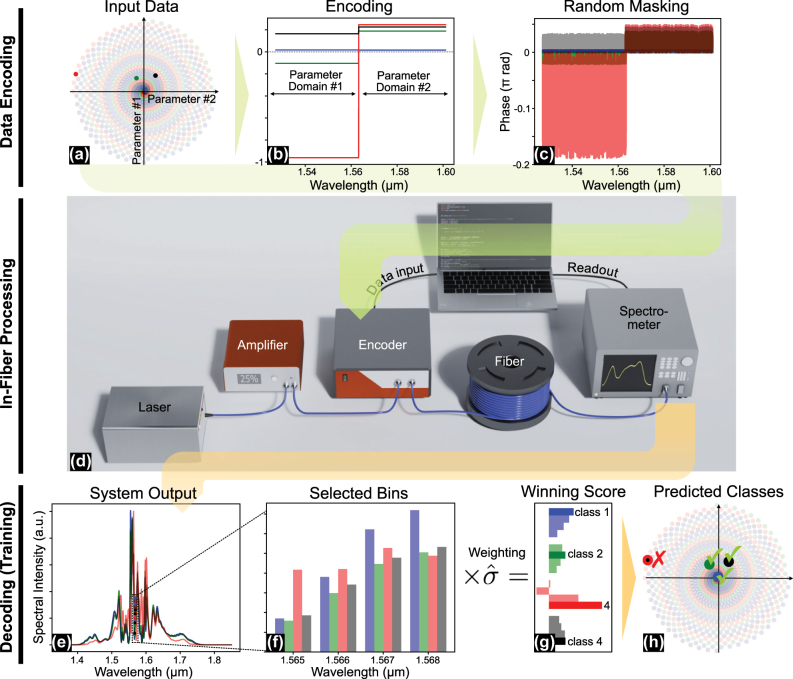
Illustration of the data flow in the fiber-based neuromorphic system using an example from the spiral dataset. (a) Input data: four sample points were selected from four different spirals. (b) Corresponding data encoding: the first half of the spectral encoding range (limited by the WaveShaper) encodes the *X*_1_-coordinate of a data tuple; the second half encodes the *X*_2_-coordinate. (c) Encoding phase after multiplying it with a constant phase scale factor and an arbitrary but fixed mask. (d) The experimental setup used for processing. A computer is used as I/O device and is not part of a feedback loop. (e) Linear spectral intensities at fiber output corresponding to the four sample inputs. (f) Linear spectral intensities at the selected, optimized search bins serving as system read-outs. (g) Prediction scores obtained by multiplying the read-outs with the trained weight matrix. Per sample, scores are sorted from class 1 to 4, from top to bottom. The highest values in a vector of four (i.e., argmax(**Y** ^score^)) determine the predicted class. (h) Prediction results: points represent the predictions, while circles around these points indicate the true class labels, the examples contain one misclassification indicated by the red cross.

We conducted experiments using a fiber-optical extreme learning machine in the frequency domain. The experimental system (see Figure 1d) comprises a femtosecond laser source (Toptica DFC) operating at 80 MHz repetition rate with 70 nm bandwidth centered at 1,560 nm, followed by an erbium-doped fiber amplifier (Thorlabs EDFA300P) operated at 25 % pump current to compensate for system losses. Data are encoded using a polarization-maintaining programmable spectral filter (Coherent Waveshaper 1000A/X) operating in the extended C- to L-band (1,528–1,602 nm), which allows to independently modify spectral phase and amplitude across 400 frequency channels for both dispersion compensation and phase information imprinting. Placing the Waveshaper after the EDFA enables precise controlling of the attenuation, and, thus, the input power to the nonlinear fiber circumventing gain saturation effects and nonlinear deterioration of the encoded phase information before being processed. The initially chirped pulses are precompressed through a dispersion-compensating fiber (Thorlabs PMDCF, 1 m length) to 400 fs before entering the spectral filter. A particle swarm optimization algorithm in conjunction with optical autocorrelation measurements has been utilized to further optimize the input pulse phase to obtain 135 fs [22], [23] before entering the main processing unit – a highly nonlinear fiber. Two nonlinear fibers were in use: (1) Thorlabs HN1550, 5 m length with all-normal dispersion (−1 ps/nm/km @1,550 nm), and 10.8 W^−1^km^−1^ nonlinear coefficient, and (2) Thorlabs PMHN1, 5 m length, with anomalous dispersion (+1 ps/nm/km @1,550 nm), and 10.7 W^−1^km^−1^ nonlinear coefficient. The output is measured using an optical spectral analyzer (Yokogawa AQ6375E) and analyzed on a standard office computer. All components are fiber-coupled and polarization-maintaining, ensuring stable operation in a compact footprint.

We illustrate how the fiber-optical ELM processes information with the example of a four-arm spiral dataset. For the spiral classification benchmark, the inputs consist solely of two coordinates for each data point, with each coordinate tuple assigned to one point of the four spirals.

Further details about the dataset are provided in the results section. The coordinate tuples are encoded into the spectral phase of a femtosecond pulse using a encoded signal, where the first half of the encoded signal corresponds to the *X*_1_-coordinate and the second half corresponds to the *X*_2_-coordinate, as illustrated in Figure 1b. To enhance the system’s sensitivity to the encoded inputs, the encoded signal is multiplied by a random mask, shown in Figure 1c. This approach is common for optical reservoir computers [24], [25], [26], [27], as it leverages the system’s sensitivity to phase jumps rather than absolute phase, ensuring a maximized system response. As a result, the output signals corresponding to different encodings become more distinguishable.

### 2.2 Supervised offline training

Following the principles of extreme learning machines [20], we train only the output layer. This method leverages the fiber-optical ELM’s inherent ability to perform complex, high-dimensional transformations efficiently, reducing the computational burden associated with traditional training. Hence, to translate the output spectra of the system into an interpretable inference, we train a weight matrix based on selecting a subset from all wavelength recordings. From this chapter onward, we call the locations of the wavelength windows “search bins” and their corresponding intensities “read-outs.” We emphasize that only a subset of the spectral outputs is used to avoid overfitting (cf. Figure 3c, ND case from 50 bins and above). Also, using only a low number of “read-outs” is taking a further step toward an all-optical neural network, as considering fewer outputs eases the practical implementation of an optical weight bank.

We select a user-defined number of search bins *n* using an optimization algorithm called *Equal Search*, a straightforward method that identifies combinations of equally spaced search bins, as described in ref. [17]. From the resulting combinations, we choose the one that minimizes the mean square error ( $\text{MSE}=\frac{1}{N}\sum {\left(WX-Y\right)}^{2},N$ is the number of test data while applying cross validations) over five cross-validations. The weight matrix *W* (*R* ^*m*^ × *R* ^*n*^ with *m* output classes and *n* search bins) is calculated using solely linear regression, as given by the closed-form solution

(1) $$W={\left(X^{T}X\right)}^{-1}X^{T}Y$$

where **X** is the read-outs vectors (intensities at selected search bins of dimension *R* ^*n*^ × *R* ^*k*^ with *k* samples), and **Y** is the task’s label vectors (ground truth of dimension *R* ^*m*^ × *R* ^*k*^). We experimented with incorporating the ridge term while training the system; however, we found it consistently yielded worse results (e.g., see long-term measurements in Figure 2A in the Supplementary Material). We assume that sampling the output bins in combination with our *Equal Search* keeps model complexity low and effectively reduces feature collinearity in the covariance matrix while maintaining only the most informative spectral features, rendering regularization unnecessary and even counterproductive.

After training the weight matrix, classification is performed by acquiring the output values at the best combination of search bins identified before. These values are stored in a vector and multiplied by the weight matrix, producing another vector, referred to as the prediction score **Y** ^score^ = **WX**. A winner-takes-all decision, i.e., **Y** ^class^ = argmax(**Y** ^score^) is then performed on the Prediction Score vector, classifying the test data based on the index of the highest score, as shown in Figure 1g and h. We compare systems based on task-specific classification accuracy in percent (%), which is defined by the ratio of number of correct class predictions over the total number of samples.

**Figure 2:**
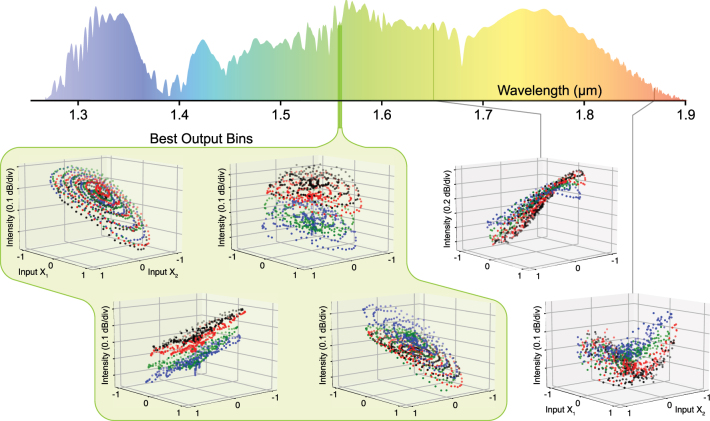
3D plots illustrating the relationship between output spectral intensity of a supercontinuum from an anomalous dispersive fiber and the input coordinates for all given samples. (a–d) Logarithmic spectral intensity versus input coordinates (X1, X2) at the optimized, selected search bins, demonstrating the system’s intrinsically distinguishable response to different classes. (e–f) Spectral intensity versus input coordinates at two randomly selected wavelength windows. All data shown are >10 dB above the spectrometer’s noise floor.

Figure 2 illustrates the nonlinear projection capabilities of our fiber-optical neural network through measured spectral response to input data tuples (*X*_1_, *X*_2_) sampled from four distinct classes in a two-dimensional coordinate system. The *Equal Search* algorithm identified four optimal search bins (Figure 2a–d) demonstrating highly distinct separation between the data classes in the spectral domain. Particularly noteworthy are the projections shown in Figure 2b and c, where the nonlinear fiber transformation achieves nearly ideal plane-wise separation of the classes in three dimensions, providing clear evidence of the system’s powerful separation capabilities. In contrast, Figure 2e and f presents randomly selected wavelength windows where the class separation is poor or nonexistent, with data points from different classes showing significant overlap.

This behavior effectively showcases the fundamental principle proposed by Huang et al. in their original ELM framework: ELMs can be understood as arbitrary kernel machines that, in optimal cases, provide hidden nodes with kernel response functions capable of transforming complex problems into linearly separable ones [20]. Following this interpretation, the performance of an ELM improves with access to a greater variety of kernel functions – a key insight that leads to our hypothesis that physical systems exhibiting richer nonlinear dynamics should outperform those with weaker nonlinearity in classification tasks.

## 3 Results and discussion

### 3.1 Scalable nonlinear classification

In this section, we demonstrate the capability of a fiber-optical ELM to effectively handle highly nonlinear tasks, exemplified by the spiral classification benchmark. The spiral classification task has emerged as a critical benchmark in the machine learning community [28] for evaluating neural networks’ capability to handle tangled data points and to learn nonlinear decision boundaries. This two-dimensional dataset, featuring interleaved spiral arms over an angular span of 2*π*, is intrinsically not linearly separable and poses a particular challenge for optical computing systems due to the inherent difficulty in implementing nonlinear activation functions in the optical domain. However, recent breakthroughs have demonstrated significant progress in this area, with recent notable achievements including on-chip complex-valued neural networks reaching 95 % accuracy on a 2-arm spiral classification [29] and a three-layer optoelectronic neural network achieving 86 % accuracy on a 4-arm spiral classification [30].

Building upon these advances, we introduce an enhanced version of this benchmark that increases the hardness of the task. We generalized the spiral dataset by a free parameter, called the maximum angular span *θ*_max_, which controls the complexity of the spiral benchmark, making it particularly effective for illustrating the impact of the network depth and the system’s nonlinear dynamics on the inference capabilities of digital and optical neuromorphic systems, respectively, as later shown in Figures 5a and 6. As such, it is an excellent general measure of the nonlinear inference capacity of new neuromorphic physical substrates.

The data points are generated using analytic equations (Eq. (2)), allowing flexibility in defining the number of phase-shifted spiral arms (number of classes, controlled by index *k*) and their wraps around the center (controlling the complexity of the task). The number of wraps defined by a full 360° turn around the center is controlled by *θ*_max_, where the number of wraps equals *θ*_max_/2*π*. For the presented results, we generated 250 points per spiral, yielding 1,000 total points across four spirals. From this set, we used 800 randomly selected points (200 per spiral) to train the system and kept 200 points (50 points per spiral) for testing after training (4:1 data split ratio).

(2) $$\begin{array}{c} X_{1}=\frac{\theta }{\theta_{\text{max}}}\cos\left(\theta +k\frac{\pi }{2}\right)\;\theta \in \left[0,\theta_{\text{max}}\right]\\ X_{2}=\frac{\theta }{\theta_{\text{max}}}\sin\left(\theta +k\frac{\pi }{2}\right)\;k\in \left\{0,1,2,3\right\} \end{array}$$

**Figure 3:**
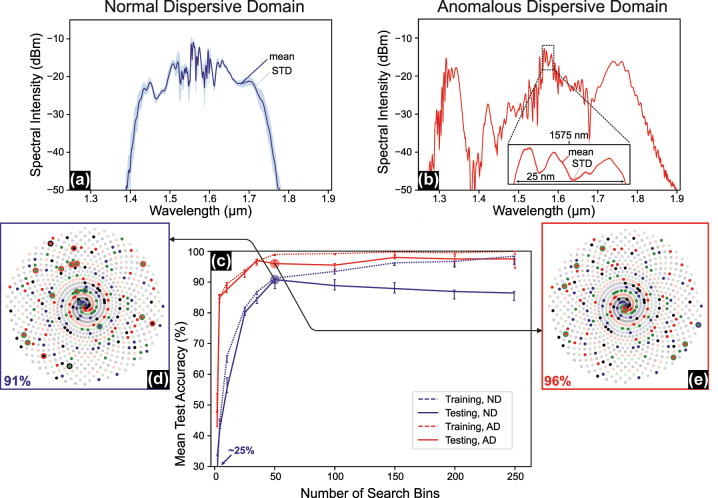
4-arms spiral classification benchmark in normal and anomalous dispersion fibers. (a) Average and standard deviation (STD) of output spectral intensities for the spirals dataset in the normal dispersion (ND) case. (b) Average and STD of output spectral intensities for the spirals dataset in the anomalous dispersion (AD) case. (c) Classification test accuracy across all classes achieved for both fiber types as a function of the number of search bins. (d) Classification results for the ND case using 50 search bins. (e) Classification results for the AD case using 50 search bins.

We evaluated our fiber-optical ELM on the spiral benchmark, with its increasing nonlinearity as *θ*_max_ increases (e.g., 0.5*π* → 10*π*), which is an ideal task to demonstrate the fiber-optical ELM’s ability to handle highly nonlinear problems. Figure 3 illustrates the classification results for *θ*_max_ = 10*π*, corresponding to 5 wraps around the center per spiral. Figure 3a and b shows the average and the standard deviation (STD) of the spectral intensities for both fiber types, normal dispersion (ND) fiber and anomalous dispersion (AD) fiber, respectively, measured under the same excitation conditions (i.e., 18.45 mW, 136 fs, cp. Table 1). The difference in standard deviations between the two cases arises from using different scale factors in the encoding phase for the two fibers.

**Table 1:** Pulse width and peak power at the input of the highly nonlinear fiber (HNLF) for various encoding conditions (attenuation), calculated using average power and pulse width (both are measured before propagating through the fiber). The pulse width was measured using an APE autocorrelator (pulseCheck NX 50). The average of 16 scans over a range of 5 ps was fitted to a Lorentzian function to determine the pulse width.

Encoding attenuation applied (dB)	Averag power measured (mW)	Pulse width measured (fs)	Peak power calculated (W)
0	18.56	136	1,705.88
2	11.66	146	998.29
4	7.41	155	597.58
6	4.72	157.5	374.60
8	3.04	161	236.02

We chose the scale factors according to the highest classification accuracy obtained during our experiments with various scale factors (see Figure 4A in the Supplementary Material). Specifically, we use *π*/2 for the ND fiber and *π*/16 for the AD fiber, resulting in a larger STD for the ND case. Our Equal Search analysis allows us to compare the system’s performance for various numbers of search bins. In Figure 3c, we observe increasing the number of search bins increases system accuracy for both fibers. The AD fiber achieves superior performance on the spiral benchmark (>95 % accuracy for 35 bins and up) compared to ND fiber, which reaches the best accuracy of 91.5 % with 50 bins before going into overfitting (i.e., test accuracy diverges from training accuracy).

### 3.2 Handwritten digit recognition

Next, we test the system’s performance on a low nonlinear task. We identified the MNIST dataset to be suitable for this purpose as it is known to be solvable to a high degree by using linear classifiers (cf. Figure 1A). The MNIST dataset is a widely used collection of handwritten digits from 0 to 9 (i.e., 10 classes), stored as 28 × 28 pixels gray-valued images, that serves as a standard benchmark in machine learning. The dataset used in this study is sourced from the TensorFlow library (tensorflow_datasets).

We trained the system on the first 2,100 images and tested it on 300 images previously unseen by the system using both normal and anomalous dispersion fibers. To encode the MNIST images, we first crop an 18 × 18-pixel window to meet the data input limitations of the Waveshaper. The cropped images were then flattened row-wise from top to bottom into 1D signals corresponding to pixel intensities. These 1D signals were multiplied by a random mask and a phase scale factor before being added to an optimized phase profile, yielding the total encoding phase (cf. Figure 3Ad), which the Waveshaper adds on the optical pulse, hence encoding the input information, before propagating through the fiber. At the output layer, the system follows the same procedure as in the spiral benchmark. It stores the output read-outs at the newly selected search bins and translates these read-outs into an interpretable prediction score by multiplying the read-outs vector with a trained weight matrix. The highest value in the prediction score (i.e., argmax(**Y** ^score^)) determines the predicted class. Figure 3A in the Supplementary Material illustrates all input and output levels for an MNIST test image.

**Figure 4:**
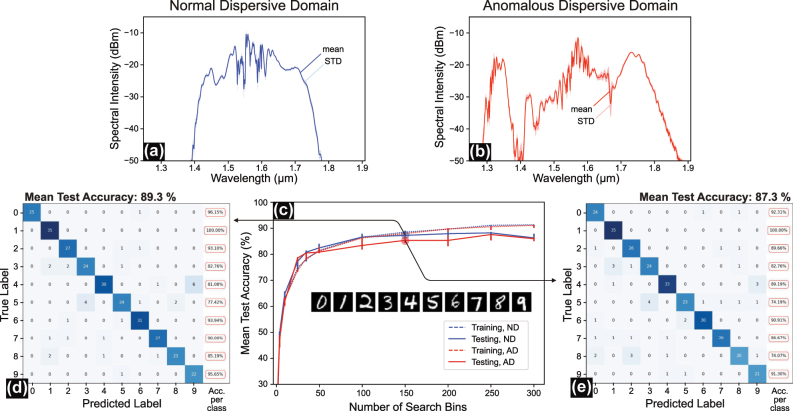
MNIST digit classification benchmark in normal and anomalous dispersion fibers. (a, b) Average and standard deviation (STD) of measured output spectral intensities for the MNIST dataset in the (a) normal dispersion (ND) case and (b) anomalous dispersion (AD) case. (c) Classification test accuracy achieved across 300 MNIST test samples across all classes as a function of the number of search bins for both fiber types. (d, e) Confusion matrices of our systems for unseen test data for the (d) ND case (achieved accuracy 89.33 %) and (e) AD case (achieved accuracy 87.3 %) using 150 search bins for both cases.

Unlike the spiral benchmark, the system demonstrates similar performance on MNIST when using ND fiber (89.33 % accuracy) and AD fiber (87.3 % accuracy), as shown in Figure 4d and e, respectively. Both cases surpass the baseline of 83.7 % for linear regression on the data [31] and perform similarly to a support vector machine (SVM) with a linear kernel, which achieves about 91 % in accuracy with only 20 support vectors (cp. Figure 1A in the Supplementary Material). Classification accuracy further improves with an increased number of search bins, as illustrated in Figure 4c.

We hypothesize that the higher nonlinear mapping introduced by the AD fiber does not significantly affect system performance for a task with low nonlinearity, such as MNIST. The slightly better performance observed with the ND fiber in our setup can be attributed to a more extensive optimization process during the selection of the optimal encoding phase scale factor. Specifically, when using the ND fiber, we conducted multiple experiments with different phase scale factors and selected the one that achieved the highest accuracy. For both fibers, a phase scale factor of $\frac{\pi }{4}$ was ultimately chosen. Figure 4a and b depicts the average spectral output intensities and their corresponding standard deviations for ND and AD fibers, respectively. The STD values in the two cases are comparable because we used the same phase scale factor.

The confusion matrices and per-class accuracy for both cases are presented in Figure 4d and e. A notable observation is the misclassification between the digit classes 3 and 5 and classes 4 and 9, which likely arises from their similar 1D intensity profiles.

To assess robustness, we conducted a long-term evaluation involving training the system on 3,000 images and testing it on 35,000 images over an acquisition period of 35 h. The system maintained an accuracy of 84.69 %, with only a modest 4.5 % drop over the extended testing period, underscoring its reliability and resilience. Further details of this robustness analysis are provided in Figure 2A in the Supplementary Material.

### 3.3 Scaling nonlinear inference capacity

We further investigate the impact of input power (consequently the system’s nonlinear mapping capability) on the performance of our fiber-optical ELM handling increasingly harder task, by changing the attenuation applied on the input signals (0, 2, 4, 6, 8 dB), while progressively increasing the spirals’ *θ*_max_ (0.5*π*, 2*π*, 4*π*, 8*π*, 10*π*) and hence the complexity of the classification task. We benchmark our system against digital neural networks with different configurations. To ensure a fair comparison between optical and digital, we fed both systems with the exact same data at a fixed training–testing split ratio.

**Figure 5:**
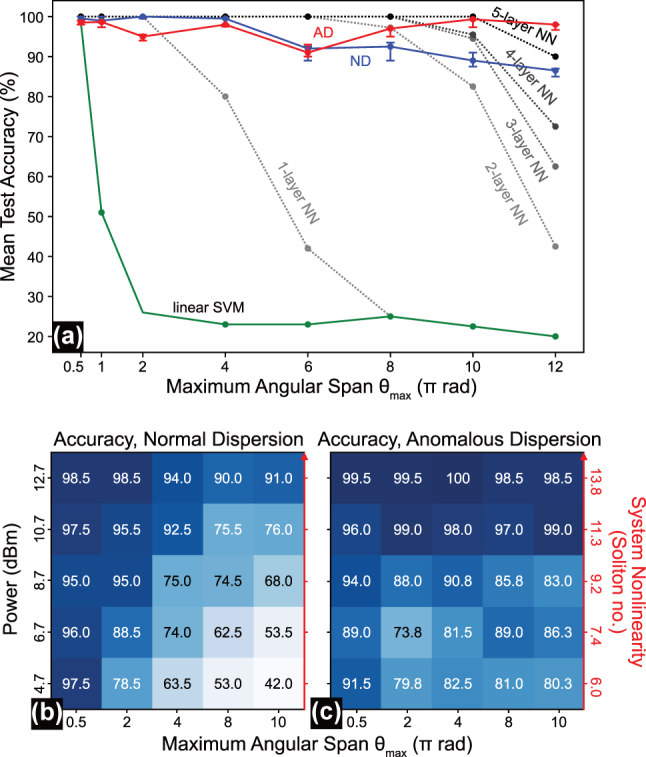
Nonlinear inference capacity scaling behavior in digital neural networks and fiber-optical extreme learning machines in two dispersive regimes. (a) Best test accuracy on 200 spiral data samples achieved by digital classifiers (a linear kernel support vector machine with 100 support vectors, and neural networks in different configurations (cp. Table 1A); all trained for 1,000 training epochs) and our fiber-optical ELM using 100 search bins for increasing nonlinear problem hardness in the spiral task, defined by the maximum angular span *θ*_max_. (b, c) Test accuracies on 200 spiral data samples as a function of system nonlinearity (or attenuation) and maximum angular span for both, (b) normal dispersion and (c) anomalous dispersion.

The results demonstrate the critical role of network depth in addressing highly nonlinear tasks with digital neural networks. As shown in Figure 5a, increasing the number of layers in deep networks, while keeping the total number of nodes constant, increases the classification accuracy of the spiral data for large *θ*_max_ significantly.

The experimental results in Figure 5a highlight the fiber-optical ELM’s exceptional efficiency in handling such highly nonlinear tasks. In particular, the AD fiber can outperform a deep network with 5 hidden layers and 1,024 nodes for *θ*_max_ > 10*π*, with the ND fiber being just a little below this performance.

Furthermore, Figure 5b illustrates the accuracy achieved using the ND fiber on the spiral benchmark with different maximum angular spans (*θ*_max_ = 0.5*π*, 2*π*, 4*π*, 8*π*, 10*π*), under different attenuation levels (0, 2, 4, 6, 8 dB), represented as rows and columns in a colormap, respectively. Figure 5c presents the corresponding results for the AD fiber. Both cases use 50 search bins.

We notice, for both dispersion domains, that applying attenuation (decreasing the input power) has a pronounced effect on system performance for highly nonlinear tasks (e.g., spirals with high *θ*_max_) but less for low nonlinear tasks (e.g., spirals with low *θ*_max_). This performance degradation with decreasing input power levels stems from the reduced variety in nonlinear system response per frequency, which forms the nonlinear mapping capability of the systems. Increasing the number of search bins mitigates the loss in nonlinear mapping induced by attenuation, which is in accordance with previous observations on comparable problems [17].

Notably, the AD fiber shows particularly better performance at lower input power levels for more complex tasks compared to the ND fiber, though with greater fluctuations. These fluctuations are most evident in the unexpectedly high accuracy for *θ*_max_ = 8*π* at attenuation of 6 dB (input power = 6.7 dBm). In particular, we observe that the AD domain features a significantly slower decline in the test accuracies of the spiral dataset with decreasing power. The AD fiber also maintains remarkably high classification accuracy as the spiral task complexity increases. Even at an angular span of 24*π* (the maximum tested in our experiments), the AD fiber system still achieves 87.5 % accuracy despite the task becoming undersampled (see Figure 5A).

We attribute the better performance of the AD fiber to the larger variety of nonlinear responses per frequency, which form the pool of functions the bin search algorithm can sample to build the nonlinear decision boundary. The richer complexity of nonlinear transformations sets the soliton fission broadening process apart from the more regulated and symmetric broadening behavior of self-phase modulation. The sensitivity of the fission process also leads to a larger variance in the test results in Figure 5c and, hence, to a less consistent trend in the decline of accuracy with system nonlinearity. Bin search techniques that allow for a more flexible positioning of the bins may reduce this effect.

It becomes apparent that the system performance depends not only on the pulse power but on the system nonlinearity in general. The system nonlinearity can be generalized by the soliton number:

(3) $$N=\;\sqrt{\frac{\gamma P_{0}T_{0}^{2}}{\left|\beta_{2}\right|}}$$

(4) $$P_{0}=\frac{P_{\text{avg}}}{T_{0}\cdot f_{\text{rep}}}$$

*γ* is the nonlinear coefficient (W^−1^km^−1^), *P*_0_ is the peak power, *T*_0_ is the measured pulse width (seconds), *β*_2_ is the dispersion coefficient (s^2^/m), and *f*_rep_ is the laser repetition rate in (Hz). Table 1 shows the power and autocorrelation measurements for the presented results.

The soliton number corresponds to a normalized nonlinear gain parameter of a waveguide system, which is proportional with peak power *P*_0_ and, hence, inversely to the attenuation level. We emphasize parametrizing the system’s nonlinear mapping capability using the soliton number rather than input power alone, as it reflects both the peak power and the pulse width of the input signal, hence the signal’s coherence, which is essential for supercontinuum generation [32].

**Figure 6:**
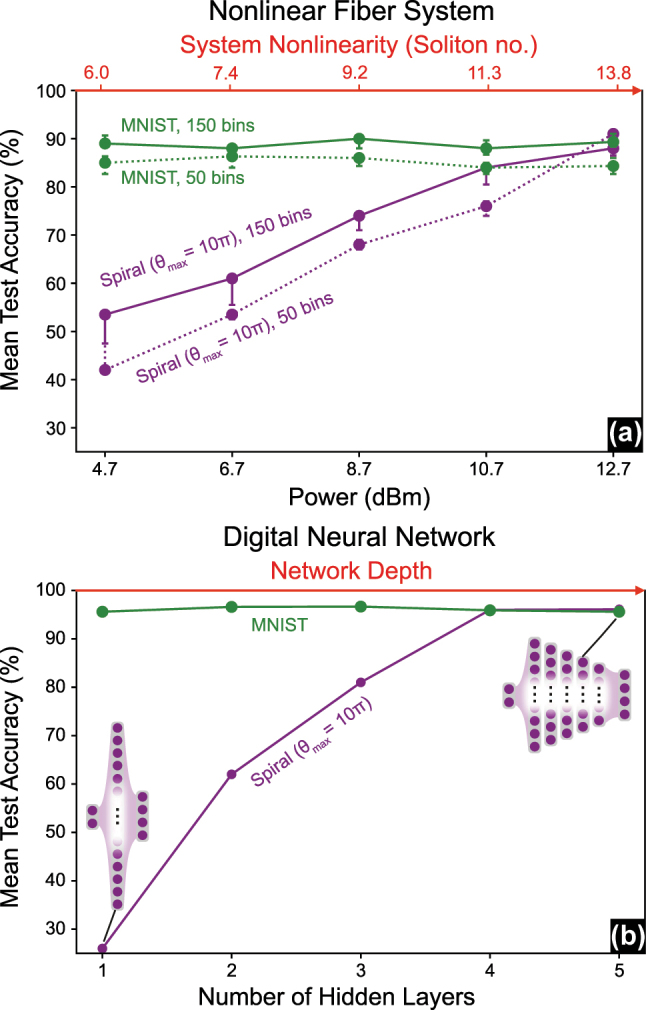
Performance comparison of optical-fiber ELM and digital neural networks handling high and low nonlinear tasks. (a) Performance trends of the fiber-optical ELM on a highly nonlinear task (spiral benchmark) and a low nonlinear task (MNIST) under varying attenuation levels. (b) Performance trends of a multilayer neural network with different numbers of hidden layers but the same total number of nodes (400) evaluated on the spiral benchmark and MNIST datasets.

Increasing the optical system’s nonlinearity (indicated by the soliton number) in an optical neuromorphic system has a similar impact on the achieved results as increasing the depth of a digital neural network. This can be seen in the similarity of the performance trends between an optical system and the digital model on a highly nonlinear task (e.g., spiral benchmark with *θ*_max_ = 10*π*) and a low nonlinear task represented by MNIST in Figure 6.

Figure 6a presents the test accuracy achieved for MNIST and the spiral benchmark (with *θ*_max_ = 10*π*) at various attenuation levels. The results are shown for the ND fiber trained using 150 search bins (solid lines) and 50 search bins (dotted lines). Increasing the system’s nonlinearity does not enhance its performance on the MNIST dataset. In contrast, higher nonlinearity significantly improves performance on the spiral benchmark with a high angular span (*θ*_max_ = 10*π*).

Notably, a similar trend is evident when solving these benchmarks using digital neural networks. Figure 6b illustrates the achieved test accuracy for deep neural networks for an increasing number of hidden layers, yet at a constant total number of nodes (400). While MNIST is solvable with 10 nodes (cf. Figure 1Aa in the Supplementary Material), a significantly higher number of nodes is needed to solve the spiral benchmark. The 400 nodes have been distributed across the hidden layers as shown in Table 2A.

The model results in Figure 6b show, as expected, that increasing the network depth has a negligible impact on the test accuracy achieved for MNIST. Here, more layers only help in convergence, enabling the model to stabilize with fewer training epochs. In contrast, consistent with the earlier results shown in Figure 5a, deeper networks provide a significant advantage when handling highly nonlinear tasks, such as the spiral benchmark with *θ*_max_ = 10*π*. Specifically, 4- and 5-hidden-layer networks with 400 nodes (see Figure 6b) outperform 2- and 3-hidden-layer networks with 1,024 nodes (see Figure 5a), highlighting the importance of network depth before connectivity when handling complex nonlinear tasks.

## 4 Conclusions

Our results suggest that while the performance of fiber-optical ELMs stalls on the MNIST dataset, they excel in handling highly nonlinear tasks. The comparable scaling behavior in classification performance between fiber ELMs regarding system nonlinearity (Figure 6a) and digital neural networks regarding the number of layers (Figure 6b) indicates that nonlinear fibers can mimic depth in neuro-inspired computing. This highlights their suitability for specialized applications where conventional optical neural networks, which are often shallow (i.e., single-layer), face limitations.

To date, the performance gain in optical neural networks is traditionally shown in improvements in image classification results across various datasets, including MNIST, fashion-MNIST, ImageNet. While these datasets are widely used, they fail to isolate the performance gains achieved through increased nonlinearity from those resulting from increased network connectivity. We demonstrated that measuring the system’s nonlinear inference capacity through scalable nonlinear benchmarks, such as the spiral dataset, provides a less ambiguous way to compare the computational depth of unconventional neuromorphic hardware. The spiral benchmark’s key strength lies in its parametric nature, allowing controlled increments in task complexity that directly test a system’s nonlinear inference capacity. This approach has proven especially valuable in two critical aspects: First, it enables quantitative comparison between different analog computing substrates, whose computational operations are often difficult to enumerate in terms of the number of performed computations. Second, it establishes a direct bridge between physical implementations and digital models, allowing us to correlate the scaling of physical system dynamics with the depth of digital neural networks. These findings may pave the way for a framework for developing standardized benchmarks that can effectively evaluate both conventional and physics-inspired computing architectures, particularly in their ability to handle increasingly complex nonlinear tasks.

We furthermore observe a direct separation of the spiral classes in selected output channels of our systems, which uniquely showcase nonlinear Schrödinger systems as genuinely arbitrary kernel machines, providing a unique substrate for implementing the ELM computing framework. The soliton number serves as a valuable, platform-independent measure of the nonlinearity as it provides a generalizable nonlinear gain parameter for wave systems that can be reduced to a nonlinear Schrödinger equation of the form

(5) $$i\partial_{Z}a+\mathrm{sgn}\left(\beta_{2}\right)\partial_{T}^{2}a=N^{2}|a{|}^{2}a$$

with complex-valued field *a*, and normalized space *Z* and time *T*. This may include mathematically similar systems such as the Korteweg–de Vries (KdV) equation (e.g., reservoirs based on shallow-water waves [33]) or the Fitzhugh–Nagumo (FN) equation used to describe membrane dynamics in nerve cells [34]. By incorporating this parameter, we gain a more nuanced understanding of the system’s behavior and its ability to process complex inputs effectively. Therefore, we hypothesize that it may provide a standardized metric for defining the nonlinear state across physical systems and comparing the nonlinear inference capacity. Nonetheless, it is important to acknowledge that it is not a universally predictive measure of a system’s computational capabilities, since those capabilities can still vary from system to system (e.g., from anomalous to normal dispersion) at the same soliton number, depending on the underlying dynamics.

Through our work, we hope to encourage ongoing exploration of generalizable performance metrics, as the connection between data-driven inference performance and the measures of intricate physical dynamics may prove vital in uncovering the genuine computational performance of natural systems. Information measures, such as Shannon Entropy [35], Fischer Information [36], or Information Processing Capacity [37], may provide a profound start, yet their application to analog feedforward ELMs remains unclear.

Our findings collectively highlight the promise of the fiber-optical Schrödinger system as a scalable, efficient solution for neuromorphic computing. Their capability to handle highly nonlinear tasks with a compact and stable design makes them a strong candidate for advancing future computing systems, in particular in a more open approach to unconventional neuromorphic computing frameworks that go beyond the perceptron architecture.

## Supplementary Material

Supplementary Material Details
