# Supplementary material for: Nonlinear inference capacity of fiber-optical extreme learning machines
Source: Nanophotonics. 2025 Jun 23;14(16):2749–60. doi: 10.1515/nanoph-2025-0045 (PMC12338873; doi:10.1515/nanoph-2025-0045)
Supplement: Supplementary file 1 — Supplementary Material Details [file j_nanoph-2025-0045_suppl_001.pdf]

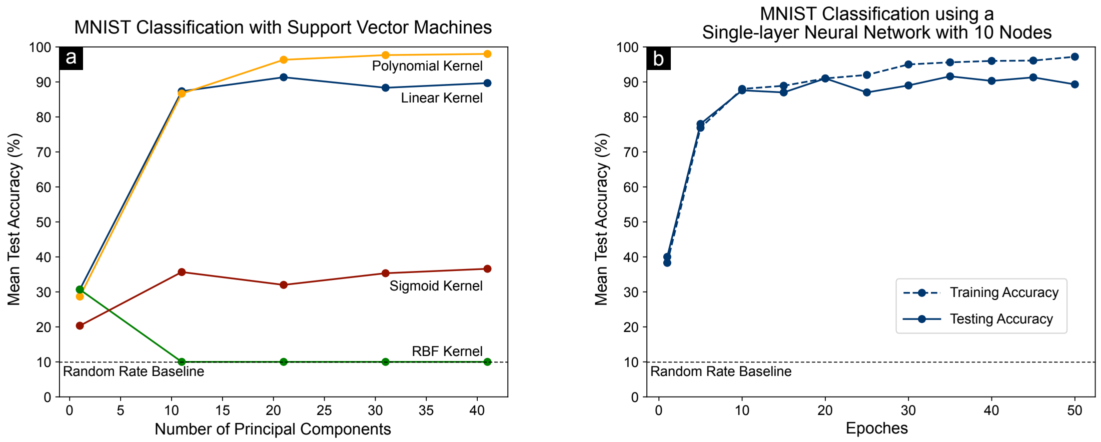

Fig. 1A: (a) Test accuracy achieved on the MNIST dataset using Support Vector Machines (SVM) with different kernels and default hyperparameters, plotted against the number of principal components used. (b) Test accuracy achieved on the MNIST dataset using a one-layer MLP with 10 hidden nodes, evaluated across different numbers of training epochs.

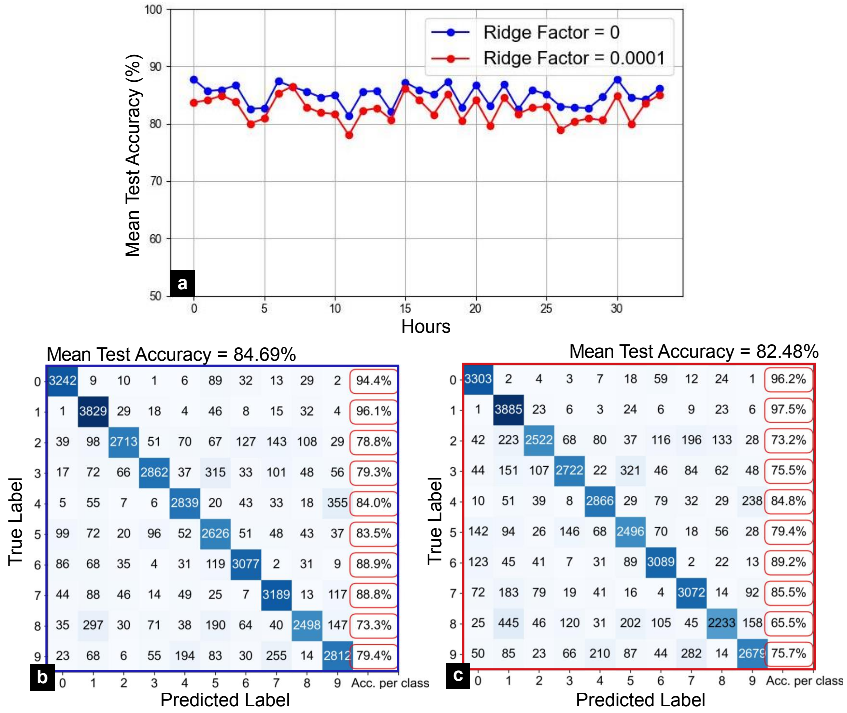

Fig. 2A: (a) Test accuracy achieved on MNIST samples recorded over a 35-hour period. Each test sample batch consists of 1000 images, while 3000 images were used for training the system, we trained the system and with and without applying a ridge factor. (b-c) Test-data confusion matrices for both cases.

Hidden layers	First layer	Second layer	Third layer	Fourth layer	Fifth layer
1	1024				
2	512	512			
3	512	256	256		
4	512	256	128	128	
5	512	256	128	64	64

Tab. 1A: First MLP, distribution of nodes (1024) across hidden layers, corresponds to the results shown in figure 5.

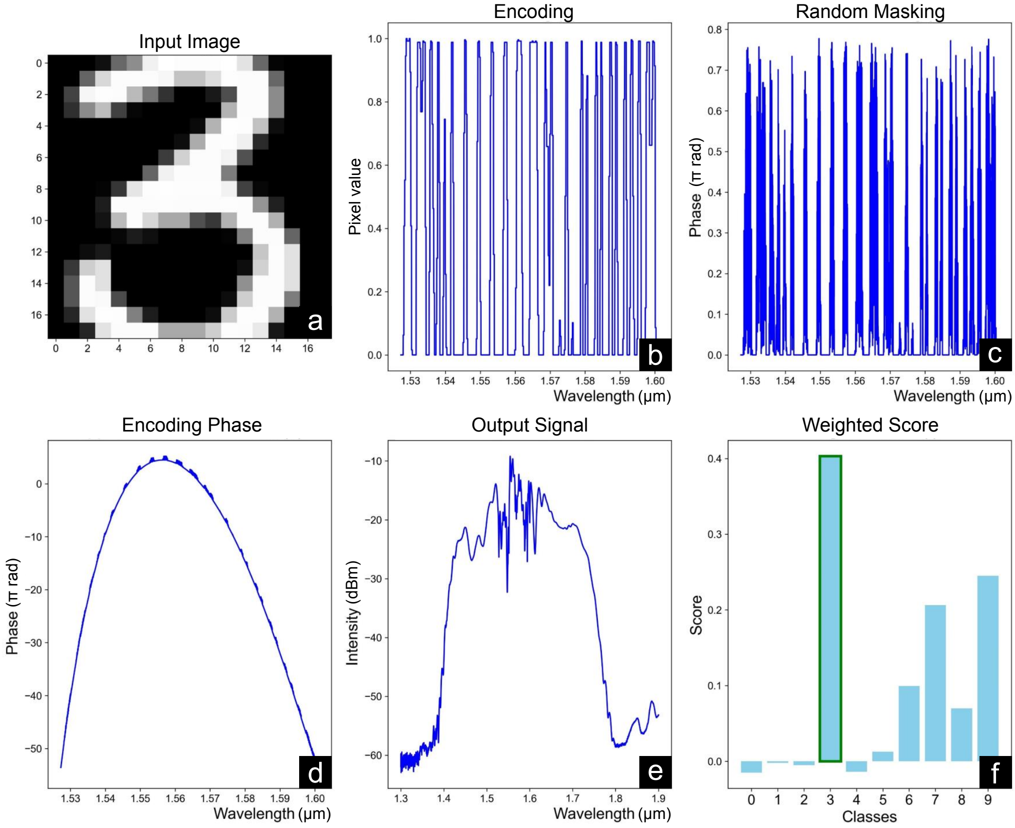

Fig. 3A: (a) Input MNIST image cropped to an 18×18-pixel window. (b) The corresponding 1D signal derived from flattening the input image. (c) The resulting encoded signal after multiplying the 1D signal by a random signal and the phase scale factor. (d) Encoding phase, generated by adding the masked signal to the optimized zero-phase profile. (e) The corresponding spectral output signal. (f) The weighted output score, obtained by multiplying the read-outs with the trained weight matrix. A winner-takes-all approach is applied, where the predicted class corresponds to the maximum score; in this case, the predicted class is "3".

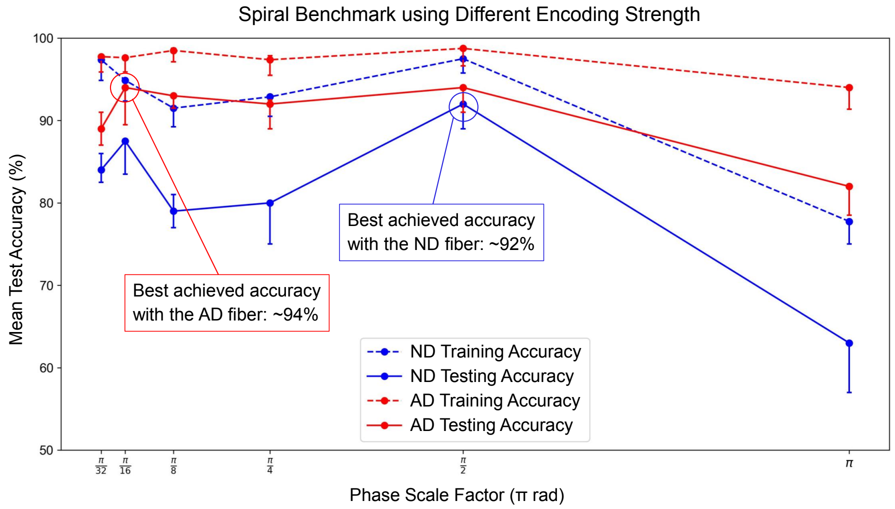

Fig. 4A: The achieved accuracy for the spiral benchmark with $\theta_{max} = 10\pi$, evaluated using different phase scale factors. All results were recorded under consistent conditions, including zero attenuation (0 dB) and identical training and testing samples.

Hidden layers	First layer	Second layer	Third layer	Fourth layer	Fifth layer
1	400				
2	200	200			
3	200	100	100		
4	200	100	50	50	
5	125	100	75	50	50

Tab. 2A: Second MLP, distribution of nodes (400) across hidden layers, corresponds to the results shown in figure 6.

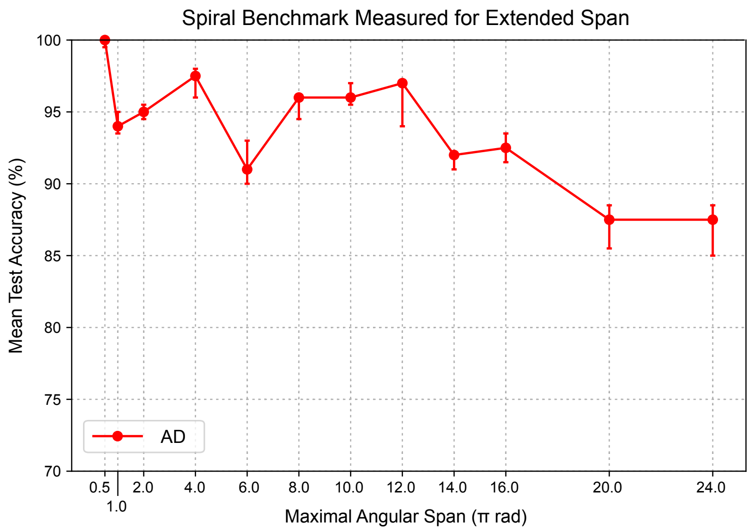

Fig. 5A: Achieved mean test accuracy on the 4-spirals dataset using an AD fiber as a processing unit vs. increasing maximum angular span of the spirals (θ_{max}) up to 24π.
